# Prognostic role of FUT8 expression in relation to p53 status in stage II and III colorectal cancer

**DOI:** 10.1371/journal.pone.0200315

**Published:** 2018-07-05

**Authors:** Masaru Noda, Hirokazu Okayama, Yasuhide Kofunato, Shun Chida, Katsuharu Saito, Takeshi Tada, Mai Ashizawa, Takahiro Nakajima, Keita Aoto, Tomohiro Kikuchi, Wataru Sakamoto, Hisahito Endo, Shotaro Fujita, Motonobu Saito, Tomoyuki Momma, Shinji Ohki, Koji Kono

**Affiliations:** 1 Department of Gastrointestinal Tract Surgery, Fukushima Medical University School of Medicine, Fukushima, Japan; 2 Department of Breast Surgery, Fukushima Medical University School of Medicine, Fukushima, Japan; 3 Department of Hepato-Biliary-Pancreatic and Transplant Surgery, Fukushima Medical University School of Medicine, Fukushima, Japan; Institut national de la recherche scientifique, CANADA

## Abstract

The expression of fucosyltransferase 8, an enzyme responsible for core fucosylation encoded by FUT8, influences tumor biology and correlates with patient prognosis in several solid cancers. We hypothesized that p53 alteration modifies prognostic associations of FUT8 expression in colorectal cancer (CRC), since FUT8 has recently been identified as a direct transcriptional target of wild-type p53. Utilizing multiple datasets of microarray and RNA sequence of CRC, FUT8 mRNA was found to be highly expressed in wild-type p53 tumors (n = 382) compared to those of mutant p53 (n = 437). Prognostic values of FUT8 expression in conjunction with the p53 status for disease-free survival (DFS) were analyzed using two independent cohorts of stage II and III CRC after curative surgery, including the immunohistochemistry (IHC) cohort (n = 123) and the microarray cohort (n = 357). In both cohorts, neither FUT8 expression nor the p53 status was associated with DFS. Strikingly, positive expression of FUT8 protein was significantly associated with better DFS only in tumors with negative p53, while it had no prognostic impact in tumors with positive p53 in the IHC cohort. Although not statistically significant, a similar prognostic trend was observed in the microarray cohort when patients were stratified by the p53 status. Our results suggest that the prognostic values of FUT8 expression on DFS may be modified by the p53 status, and the expression of FUT8 protein can be a prognostic biomarker for patients with stage II and III CRC.

## Introduction

Colorectal cancer (CRC) is one of the most common causes of cancer-death worldwide [1]. Most of CRC develops through the accumulation of various genetic alterations, including WNT signal activation, KRAS mutations, p53 inactivation and chromosomal instability. This genetic and molecular diversity can result in heterogeneous patient outcomes [2]. Surgery is the standard treatment for localized disease, however, approximately 25–40% of stage II and III patients will suffer from tumor recurrence even after curative resection. Large randomized studies revealed that postoperative adjuvant chemotherapy can improve survival in stage III patients, but it is not routinely recommended for stage II patients [3]. By contrast, a considerable fraction of those patients is likely to be cured by surgery alone, thus they might be spared from intensive postoperative surveillance or adjuvant treatment. Therefore, molecular biomarkers are needed to stratify the risk of relapse after surgery in patients with stage II and III CRC.

Fucosylation, which comprises the transfer of fucose to glycoproteins and glycolipids, is synthesized by a family of fucosyltransferases (FUTs) [4]. Aberrant fucosylation due to dysregulated expression of FUTs frequently occurs during tumor progression, involving in fundamental cellular biology processes occurring in cancer. Among 13 human FUTs that have been identified, α1,6-fucosyltransferase, encoded by FUT8, is the only enzyme responsible for core fucosylation, which catalyzes the transfer of α1,6-fucose to the innermost GlcNAc residue of N-glycans [5]. The upregulated expression of FUT8 has been reported in several cancers, including lung cancer [6], prostate cancer [7], hepatocellular carcinoma (HCC) [8–10] and CRC [11], demonstrating that FUT8 is involved in biological tumor characteristics and patient outcomes. It is worth noting that core fucosylation of alpha-fetoprotein (AFP-L3 fraction) due to the upregulation of FUT8 in HCC cells is a FDA-approved serum tumor marker for the specific diagnosis of HCC [12, 13]. Also, a recent study has demonstrated that FUT8 can be transcriptionally activated by wild-type p53, encoded by tumor suppressor gene TP53, through p53 biding to its responsive elements within the FUT8 promotor region in HCC [14]. Since p53 alterations are found frequently (~50%) not only in HCC but also in CRC and other cancer types, the expression and function of FUT8 may be modulated by p53 in human cancers. However, the association between FUT8 expression and the p53 status in CRC has not been addressed. Although a previous report by Muinelo-Romay et al. showed that FUT8 expression was associated with disease-free survival (DFS) in CRC [15], we speculated that the prognostic effect of FUT8 might be confounded by the p53 status. The present study utilized multiple transcriptional datasets based on microarrays and RNA sequence (RNA-Seq) of CRC to determine if the expression of FUT8 is associated with p53 mutations. We then tested the effect of FUT8 expression on survival outcome using two independent cohorts based on immunohistochemistry (IHC) and microarray analysis for patients with stage II and III CRC, and its prognostic values were further addressed in conjunction with the p53 alteration status.

## Materials and methods

### Microarray and RNA-sequence data analysis

All microarray data are publicly available from the Gene Expression Omnibus (GEO) database (http://www.ncbi.nlm.nih.gov/geo). The pre-processed expression values obtained from each dataset were utilized. We compiled four independent microarray cohorts, for which TP53 mutation status were available, consisting of GSE41258 [16] based on Affymetrix Human Genome UA133A platform, and GSE39582 [17], GSE39084 [18] and GSE35896 [19] based on Affymetrix Human Genome U133 Plus 2.0 platform. If a gene is represented by multiple probes, the expression values of multiple probes were averaged. For the TCGA samples, level 3 Illumina RNA-Seq data processed by the RPKM method for both colon and rectal adenocarcinoma (COADREAD) were downloaded through cBioPortal (http://www.cbioportal.org/) [20, 21]. Somatic mutations in TP53 gene were obtained from the TCGA data portal (http://tcga-data.nci.nih.gov/). To examine the expression levels of FUT8 mRNA in p53 mutant and wild-type tumors, respectively, those 5 datasets based on different platforms were analyzed using Z-scores for the expression values of FUT8 in each cohort.

For survival analysis according to FUT8 expression and the p53 status, two microarray datasets, GSE41258 and GSE39582, which had DFS information with long-term follow-up were utilized. We focused only on patients with stage II and III CRC for which the p53 status was available. The expression of FUT8 mRNA was dichotomized as low or high on the basis of the median expression value for each cohort separately (GSE41258, GSE39582-discovery set, and GSE39582-validation set), and then these cohorts were combined for further analysis.

### Colorectal cancer samples

We enrolled 318 consecutive patients with stage I to IV primary CRC, who underwent surgery between 1990 and 2010 in Fukushima Medical University Hospital as described previously [22]. Tumors were classified according to the TNM classification of malignant tumors (UICC 7th edition). Clinical information was retrospectively obtained by reviewing medical records, with the last follow-up in February 2016. For survival analysis, we used 194 stage II and III patients who underwent curative resection with available survival information. The primary endpoint of interest was DFS, which was defined as time from the date of surgery to the date of disease recurrence. The study was conducted in accordance with the Declaration of Helsinki and was approved by the Institutional Review Board of Fukushima Medical University, and granted waiver of written consent.

### Cell culture

CRC cell lines, including SW48, LS180, and Colo205 were previously obtained [23] and authenticated by Short tandem repeat (STR) analysis (Promega, Madison, WI, USA). HCT116 was obtained from JCRB Cell Bank (Osaka, Japan). LS180 cells were maintained with DMEM; others with RPMI-1640 containing 10% fetal bovine serum and penicillin/streptomycin (ThermoFisher Scientific, Waltham, MA, USA) at 37°C in a humidified atmosphere of 5% CO_2_. Cells were fixed in 10% formalin and embedded in paraffin, and then used for IHC.

### Immunohistochemistry

Primary rabbit polyclonal anti-FUT8 antibody (HPA043410, Prestige Antibodies Powered by Atlas Antibodies, Sigma-Aldrich, Co. LLC. St. Louis, MO, USA) was identified using the Human Protein Atlas database, in which the antibody reliability is scored as “Supported” according to standard IHC validation based on RNA consistency and literature conformity (www.proteinatlas.org) [24, 25]. For p53 staining, primary mouse monoclonal anti-p53 antibody (clone: DO-7, Dako, Glostrup, Denmark) was used. IHC was performed as previously described [22]. In brief, four-μm thick, whole tumor sections were deparaffinized, and then endogenous peroxidases were blocked. Antigens were retrieved by autoclave for 5 min in 10 mM citrate buffer solution (105°C, pH 6.0) for p53 staining. Diluted antibodies (1:200 for FUT8, 1:100 for p53) were incubated at 4°C overnight, and detected by a horseradish peroxidase (HRP)-coupled anti-rabbit or anti-mouse polymer followed by incubation with diaminobenzidine (Dako EnVision+ System, Dako, Heverlee, Belgium). Sections were counterstained with hematoxylin. For FUT8 staining, each sections were evaluated by two independent investigators according to the procedure as previously described [15]. Briefly, FUT8 staining pattern in tumor was initially classified as 0 (no staining), 1 (weak, less than 10% staining), 2 (moderate, 10–50% staining), and 3 (strong, more than 50%), and then tumors were divided into low (score 0, 1) or high (score 2, 3) FUT8 protein expression. The staining of p53 was evaluated as the fraction of tumor cells with moderate/strong nuclear staining for p53, and p53 positivity was defined as ≥10% of tumor cells with nuclear staining as described previously [26, 27].

### Statistical analyses

Fisher’s exact test, Chi-square test, unpaired t-test and Mann-Whitney U test were used to determine differences between two variables. Cumulative survival was estimated by the Kaplan-Meier method, and differences between the two groups were analyzed by log-rank test. Univariate and multivariate models were computed using Cox proportional hazards regression. All statistical analyses were two-sided and were conducted using Graphpad Prism v6.0 (Graphpad Software Inc., La Jolla, CA, USA) and SPSS Statistics version 24 (IBM Corporation, NY, USA). All P-values were two-sided, and P-values less than 0.05 were considered statistically significant.

## Results

### FUT8 mRNA expression was associated with the p53 status in gene expression datasets

**Fig 1:**
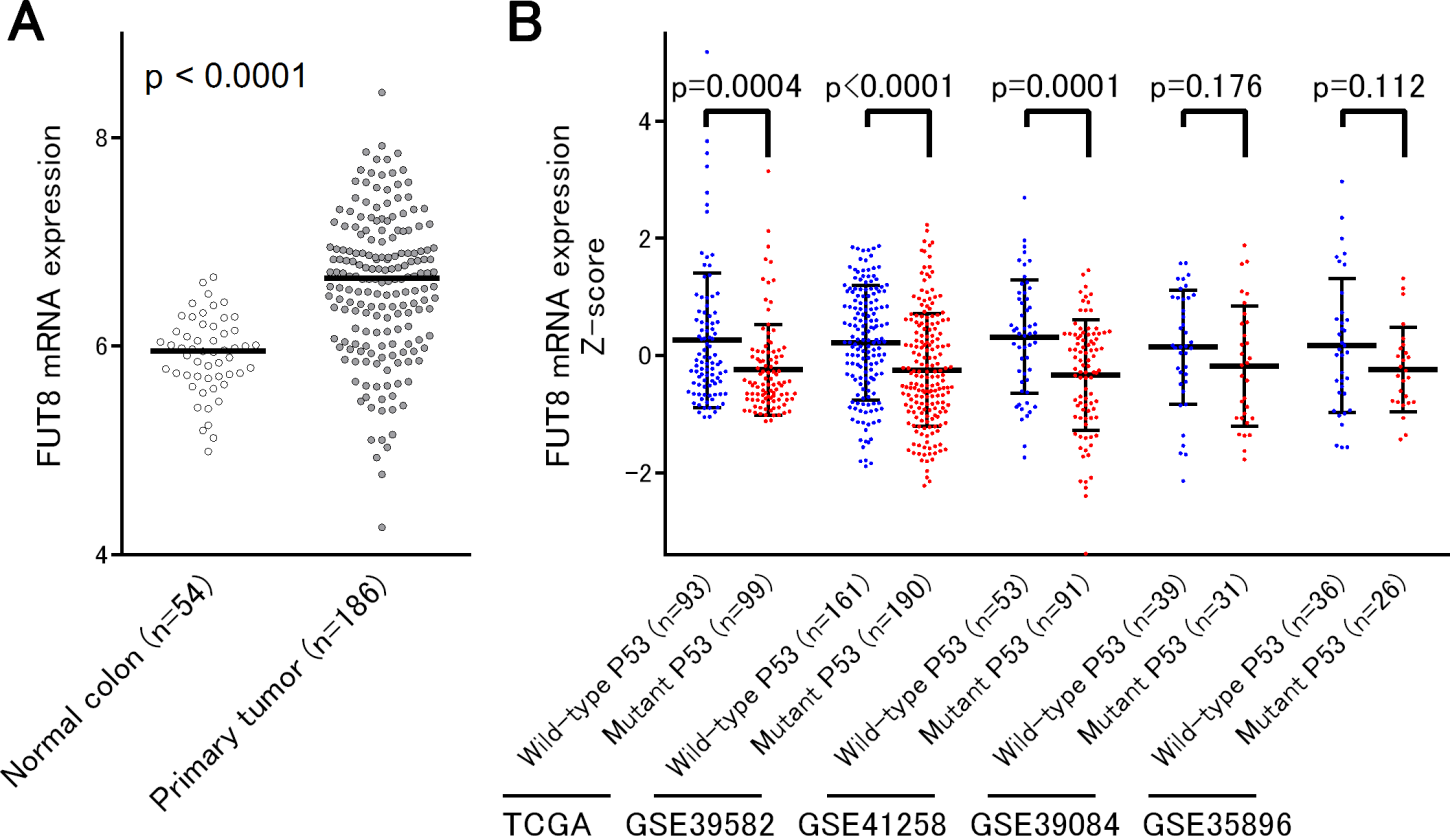
The expression of FUT8 mRNA in multiple cohorts of colorectal cancer. (A) FUT8 mRNA expression was significantly upregulated in primary tumors compared to normal colon mucosa. (B) In five independent datasets of colorectal cancer, higher levels of FUT8 mRNA expression were consistently observed in tumors with wild-type p53 than those of mutant p53.

We first analyzed the expression of FUT8 at mRNA levels in a microarray dataset GSE41258, in which expression data for normal (n = 54) and cancer tissues (n = 186) were available. This analysis clearly confirmed the previous observation that FUT8 was significantly upregulated in CRC tissues compared to normal tissues (P < 0.0001) (Fig 1A) [11]. We then attempted to determine whether the expression of FUT8 mRNA is associated with the p53 status in CRC. For this purpose, we assembled 5 independent datasets of CRC based on microarray or RNA-seq platforms, including TCGA [20], GSE41258 [16], GSE39582 [17], GSE39084 [18] and GSE35896 [19], in which p53 mutation data were reported. This enabled us to conduct a large scale association analysis of the p53 status with FUT8 expression using a total of 819 CRC patients, consisted of tumors harboring 382 wild-type and 437 mutant p53 (Fig 1B). We found that FUT8 expression levels were significantly higher in p53 wild-type tumors than tumors with mutant p53 in 3 of 5 cohorts, including TCGA, GSE39582 and GSE41258 (P < 0.001). In the 2 remaining cohorts, including GSE39084 and GSE35896, FUT8 mRNA seemed to be consistently highly expressed in p53 wild-type tumors compared to those with mutant p53, although it did not reach statistical significance probably due to the relatively small sample size in those cohorts. Those findings might seem consistent with our hypothesis that FUT8 expression can be regulated by p53, as the recent report has demonstrated that FUT8 is a direct transcriptional target of wild-type p53 [14].

### FUT8 protein expression by immunohistochemistry

**Fig 2:**
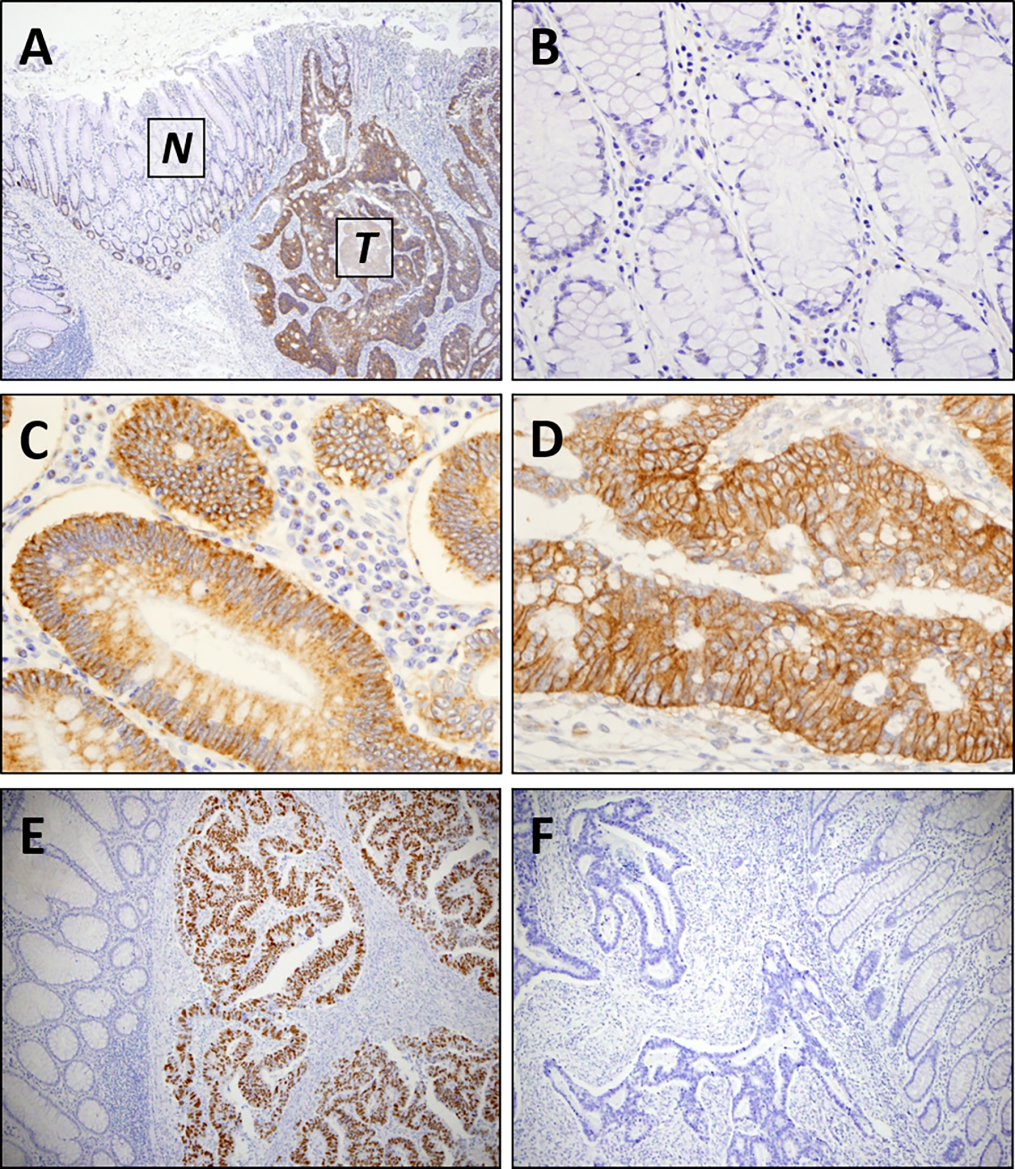
Representative images of immunohistochemistry for FUT8 and p53 expression in colorectal cancer. (A) FUT8 protein expression in colon carcinoma [T] and adjacent colon mucosa [N]. (B) FUT8 was not expressed by non-neoplastic colon mucosal cells. (C) FUT8 staining was typically found in cytoplasm of tumor cells. (D) Occasionally, concomitant cytoplasmic and membranous staining of FUT8 in tumor cells can be found. (E) p53-positive tumor showing strong nuclear staining in cancer cells. (F) p53-negative tumor showing no nuclear staining. Magnification: (A,E,F) x100; (B,C,D) x400.

To evaluate FUT8 protein expression, we initially conducted IHC using CRC cell line samples with a validated antibody supported by the Human Protein Atlas. As demonstrated in S1 Fig, cytoplasmic distribution of FUT8 staining was found in SW48, HCT116, Colo205 and LS180 cell lines, while LS180 cells showed both cytoplasmic and membranous immunoreactivity. Correspondingly, in human CRC specimens, FUT8 staining was primarily located within cytoplasm of tumor cells, which was occasionally accompanied by tumor cell membranous staining (Fig 2A–2D and S2 Fig). Those findings were highly consistent with the staining patterns demonstrated in the Human Protein Atlas. In contrast to the tumor cells, the vast majority of adjacent mucosal cells lacks FUT8 staining, consistent with previous IHC studies for FUT8 in CRC [11, 15], although some normal mucosal cells in the basal layer tended to exhibit weak/equivocal staining (Fig 2A and 2B and S2 Fig).

Given the consistent association between FUT8 expression and the p53 status at least in transcriptional levels, we conducted IHC for FUT8 protein expression in 318 primary CRC specimens (Fig 2A–2D). In this IHC cohort, 196 tumors had p53 alteration status determined by nuclear accumulation of p53 protein by IHC (Fig 2E and 2F). Among 318 patients with stage I to IV CRC, high expression of FUT8 was observed in 248 (78.0%) tumors. However, none of the clinicopathological parameters, including age, gender, tumor location, histological differentiation, TNM staging, was associated with FUT8 protein expression in our cohort (Table 1). In addition, unlike mRNA expression levels, no association was found between FUT8 protein expression and the p53 status (Table 1).

**Table 1:** Clinicopathological characteristics of colorectal cancer patients according to FUT8 expression.

		Total (n = 318)	FUT8 protein expression				
			Low		High		*P*
			n = 70 (22.0%)		n = 248 (78.0%)		
Age	Mean±SD	67.0±11.7	65.6±12.0		67.3±11.7		0.273
Gender							0.494
	Male	188	44	(62.9%)	144	(58.1%)	
	Female	130	26	(37.1%)	104	(41.9%)	
Tumor location							0.363
	Proximal colon	100	25	(35.7%)	75	(30.2%)	
	Distal colon	97	21	(30.0%)	76	(30.6%)	
	Rectum	121	24	(34.3%)	97	(39.1%)	
Histological differentiation							0.705
	Well	152	37	(51.4%)	116	(46.8%)	
	Moderately	152	29	(41.4%)	123	(49.6%)	
	Poorly	14	5	(7.1%)	9	(3.6%)	
Stage (UICC)							0.821
	I	62	16	(22.9%)	46	(18.5%)	
	II	122	22	(31.4%)	100	(40.3%)	
	III	89	25	(35.7%)	64	(25.8%)	
	IV	45	7	(10.0%)	38	(15.3%)	
Tumor invasion							0.388
	T1	33	6	(8.6%)	27	(10.9%)	
	T2	49	14	(20.0%)	35	(14.1%)	
	T3	138	32	(45.7%)	106	(42.7%)	
	T4	98	18	(25.7%)	80	(32.3%)	
Lymph node metastasis							0.211
	Absent	196	39	(55.7%)	157	(63.3%)	
	Present	119	31	(44.3%)	88	(35.5%)	
	Not available	3	0	(0.0%)	3	(1.2%)	
Distant metastasis							0.333
	Absent	273	63	(90.0%)	210	(84.7%)	
	Present	45	7	(10.0%)	38	(15.3%)	
p53 immunohistochemistry							1.000
	Negative	100	24	(34.3%)	76	(30.6%)	
	Positive	96	24	(34.3%)	72	(29.0%)	
	Not available	122	22	(31.4%)	100	(40.3%)	

### Association of FUT8 expression with DFS in stage II and III CRC

**Fig 3:**
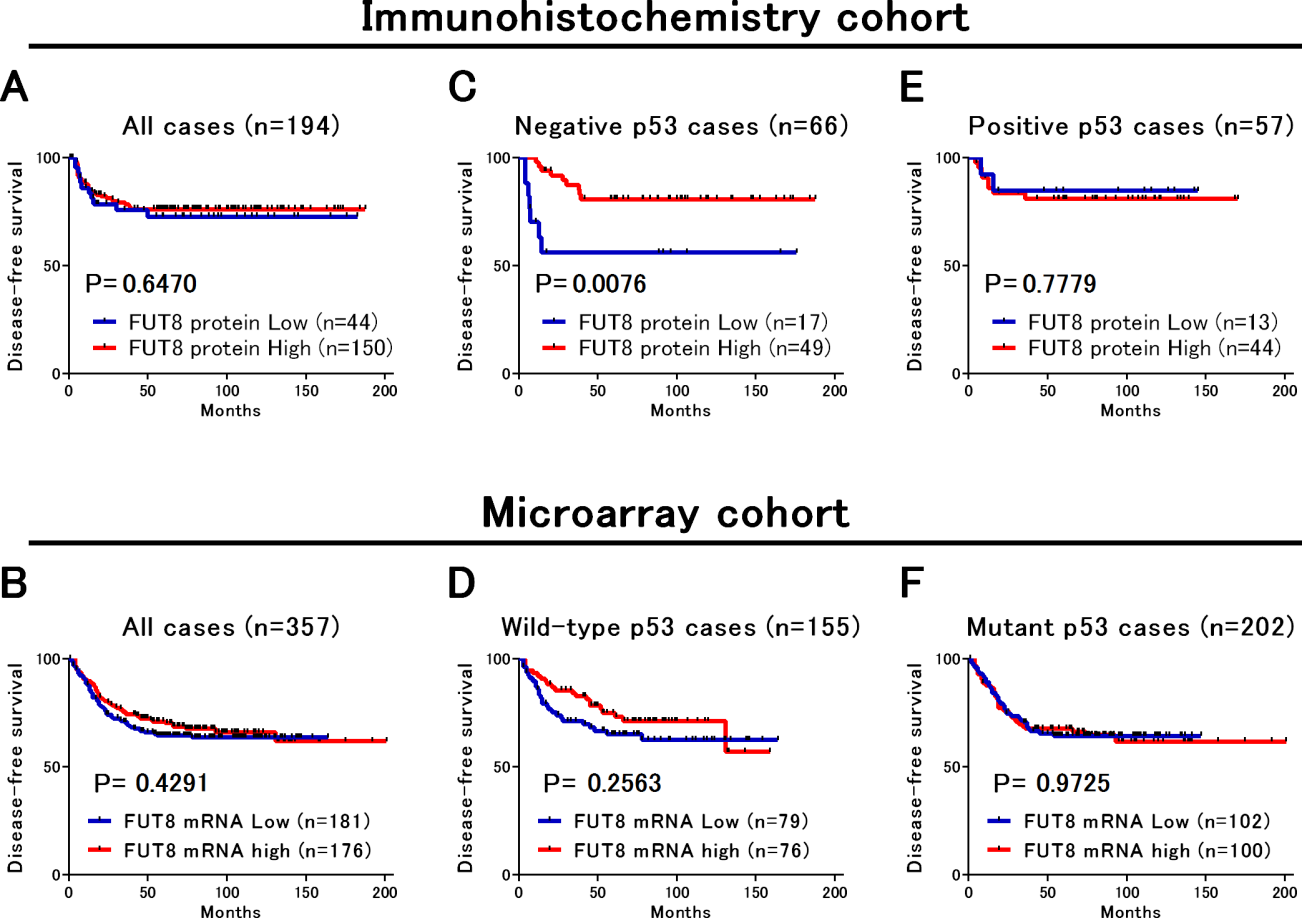
Disease-free survival in two cohorts of stage II and III colorectal cancer. (A,B) FUT8 protein or mRNA expression had no significant impact on survival in both cohorts. (C) In patients with p53-negative tumors, FUT8 protein expression was significantly associated with better survival in the IHC cohort. (D) In patients with wild-type p53 tumors, FUT8 mRNA expression tended to be associated with better survival in the microarray cohort. (E,F) In patients with p53-positive tumors or mutant p53 tumors, FUT8 protein or mRNA expression showed no association with survival.

We next examined the relationship between FUT8 protein expression and survival in 194 patients with stage II and III CRC who underwent curative surgery. However, FUT8 protein expression was not associated with DFS in Kaplan-Meier analysis (Log-rank; P = 0.647) and in univariate and multivariate Cox analysis [univariate hazard ratio (HR) = 0.85, 95% confidence interval (CI) = 0.43 to 1.69, multivariate HR = 0.57, 95%CI = 0.25 to 1.31] (Fig 3A, Table 2 and S1 Table). Also, positive p53 expression had no impact on DFS (HR = 0.72, 95%CI = 0.33 to 1.59). To further address the prognostic significance of FUT8 expression, we utilized microarray datasets for survival analysis. Two independent datasets, including GSE41258 and GSE39582 in which the p53 status as well as DFS information were available, were combined to increase statistical power, and FUT8 mRNA expression was divided into low or high based on the median expression value within each cohort. The microarray cohort consisted of a total of 357 patients with stage II and III CRC, including 155 wild-type and 202 mutant p53 tumors. Similar to the analysis of the IHC cohort, no association was found between FUT8 mRNA expression and DFS in Kaplan-Meier analysis (Log-rank; P = 0.429) and in univariate and multivariate Cox analysis (univariate HR = 0.86, 95%CI = 0.60 to 1.24, multivariate HR = 0.86, 95%CI = 0.59 to 1.24) (Fig 3B, Table 2 and S1 Table).

**Table 2:** Univariate and multivariate Cox regression analysis for disease-free survival in patients with stage II and III colorectal cancer.

		N	Univariate			Multivariate †		
			HR	95%CI	*P*	HR	95%CI	*P*
Immunohistochemistry cohort (n = 194)								
	FUT8 protein Low	44	1 (Reference)			1 (Reference)		
	FUT8 protein High	150	0.85	0.43–1.69	0.649	0.57	0.25–1.31	0.188
Microarray cohort (n = 357) ‡								
	FUT8 mRNA Low	181	1 (Reference)			1 (Reference)		
	FUT8 mRNA High	176	0.86	0.60–1.24	0.418	0.86	0.59–1.24	0.412

HR, hazard ratio; CI, confidence interval.

† The multivariate models were adjusted for variables that were significant in the univariate analysis.

‡ In the microarray cohort, univariate and multivariate models were also adjusted for cohort membership.

### FUT8 protein expression was associated with better survival in patients with p53-negative CRC

To test our main hypothesis, we examined the association of patient survival with FUT8 expression according to the p53 status in Kaplan-Meier analysis (Fig 3C–3F) and univariate and multivariate Cox analysis (Table 3). By stratifying patients on the basis of the p53 status in the IHC cohort, we found differential prognostic effect of FUT8 protein expression according to the p53 status, demonstrating that high FUT8 protein expression was significantly associated with better DFS only among patients with p53-negative tumor (Log-rank; P = 0.0076, Fig 3C), but not among patients with p53-positive tumor (Log-rank; P = 0.7779, Fig 3E). As shown in Table 3, univariate and multivariate Cox analysis revealed that high FUT8 protein expression was significantly and independently associated with better DFS only among patients with p53-negative tumors (univariate HR = 0.28, 95%CI = 0.10 to 0.76, multivariate HR = 0.31, 95%CI = 0.11 to 0.88). By contrast, in patients with p53-positive tumor, FUT8 had no significant influence on DFS in both univariate and multivariate analysis (univariate HR = 1.25, 95%CI = 0.27 to 5.88, multivariate HR = 1.47, 95%CI = 0.30 to 7.22). We further attempted to examine whether the influence of FUT8 mRNA expression on DFS was modified by the p53 status in the microarray cohort. Although not statistically significant, patients with high FUT8 mRNA expression tended to have better prognosis only when wild-type p53 tumors were analyzed (Log-rank; P = 0.2563, Fig 3D), while this tendency was not present among p53 mutant tumors (Log-rank; P = 0.9725, Fig 3F). Univariate and multivariate Cox analysis demonstrated that FUT8 mRNA was not significantly associated with DFS among tumors with wild-type p53 (multivariate HR = 0.74, 95%CI = 0.42 to 1.31) or those with mutant p53 (multivariate HR = 1.00, 95%CI = 0.63 to 1.61).

**Table 3:** Univariate and multivariate Cox regression analysis for disease-free survival in patients with stage II and III colorectal cancer according to FUT8 expression and the p53 status.

			n	Univariate			Multivariate †		
				HR	95%CI	*P*	HR	95%CI	*P*
Immunohistochemistry cohort (n = 123)									
	p53 negative								
		FUT8 protein Low	17	1 (Reference)			1 (Reference)		
		FUT8 protein High	49	0.28	0.10–0.76	0.012	0.31	0.11–0.88	0.027
	p53 positive								
		FUT8 protein Low	13	1 (Reference)			1 (Reference)		
		FUT8 protein High	44	1.25	0.27–5.88	0.778	1.47	0.30–7.22	0.634
Microarray cohort (n = 357) ‡									
	p53 wild-type								
		FUT8 mRNA Low	79	1 (Reference)			1 (Reference)		
		FUT8 mRNA High	76	0.72	0.41–1.28	0.261	0.74	0.42–1.31	0.303
	p53 mutant								
		FUT8 mRNA Low	102	1 (Reference)			1 (Reference)		
		FUT8 mRNA High	100	1.02	0.64–1.64	0.926	1.00	0.63–1.61	0.991

HR, hazard ratio; CI, confidence interval.

† The multivariate models were adjusted for variables that were significant in the univariate analysis.

‡ In the microarray cohort, univariate and multivariate models were also adjusted for cohort membership.

## Discussion

Aberrant fucosylation and dysregulation of FUTs have been frequently found in human cancer [4, 5]. Fucosylated glycoproteins recognized by monoclonal antibodies have widely been used as serum tumor markers, including sialyl Lewis a (sLe^a^, known as CA19-9) and sialyl Lewis x (sLe^x^, known under the common names of SLX or NCC-ST-439) for monitoring of many cancer types, including CRC [12, 28, 29]. Among 13 FUTs, α1,3- and α1,4-fucosyltransferases, including FUT3, 4, 5, 6, 7 and 9, are involved in the biosynthesis of Lewis and sLe epitopes [4, 5]. Also, FUT7 overexpression induced by hypoxia is involved in abnormal sLe^x^ and sLe^a^ synthesis [28], while FUT6 has been reported as a key regulator of sLe^x^ biosynthesis in CRC [30]. Unlike other FUTs, only FUT8 can catalyze α1,6-fucosylation (core fucosylation) that are particularly involved in a variety of physiological processes and in cancer biology [4, 12]. Importantly, FUT8 has been known to be upregulated in several types of human cancer, including HCC and CRC, and is known to be responsible for the synthesis of HCC-specific serum tumor marker, AFP-L3.

In response to a variety of cellular stresses, p53 as a transcription factor binds specific promoters to regulate gene expression that drive many biological processes, including cell-cycle arrest, apoptosis, senescence, exerting its tumor suppressive functions. Inactivation of p53 is one of the most common events in carcinogenesis, with approximately 50% of human cancer, including CRC, carries p53 mutations. The cancer-associated p53 mutations are primarily missense substitutions that cause single amino-acid changes, resulting in the loss of wild-type functions and also exerting a dominant-negative regulation over the remaining wild-type p53 in most cases. Such missense mutations frequently lead to stabilization of p53 protein that accumulates in tumor nuclei, which can be detected by IHC as a surrogate marker for mutation status [31].

In this study, the p53 status was determined by somatic mutation analysis in the microarray cohort or immunohistochemical detection by the IHC cohort. Both of these cohorts showed that the p53 status itself had absolutely no impact on DFS. This is not surprising, since the prognostic significance of the p53 status continues to be one of the most controversial areas of p53 research in human cancer [32]. Also, in CRC, it has still been unclear if p53 abnormalities based on mutation analysis or IHC can be markers of survival outcomes [33, 34].

In the present study, we specifically focused on FUT8, with the initial aim to examine the prognostic value of FUT8 expression on DFS in patients with stage II and III CRC after curative surgery. Recently, FUT8 has been identified as a direct transcriptional target of wild-type p53 at least in HCC cells, suggesting that the p53 status can affect the expression and function of FUT8. Thus, we further addressed the hypothesis that the prognostic effect of FUT8 expression may differ by the p53 status. Firstly, using a large combined cohort of CRC, consisting of 819 tumors that were obtained from 5 independent datasets of high-throughput gene expression analysis, we found for the first time that FUT8 mRNA expression was significantly higher in p53 wild-type tumors compared to those with mutant p53. This suggests that alterations of p53 can cause dysregulation of FUT8 mRNA levels possibly through transcriptional mechanism. As the FUT8 promotor region is likely to carry the responsive element of wild-type p53 [14], loss of wild-type p53 function due to TP53 mutations might be responsible for the altered FUT8 mRNA expression. However, this finding was not confirmed in FUT8 protein levels, in which no association was found between FUT8 expression and abnormal p53 expression by IHC. Thus, future studies would be required to mechanistically address the functional relevance of p53 alteration to regulating transcription and translation of FUT8. Most recently, two studies have revealed that wild-type p53 can transcriptionally activate FUCA1, a fucosidase gene, in CRC cells by direct binding to its responsive element [35, 36]. On the other hand, fucosyltransferase 3, encoded by FUT3, was found to be upregulated in CRC harboring p53 mutations compared to tumors with wild-type p53 [37]. Intriguingly, a serum glycome study of breast cancer demonstrated that core-fucosylated glycans, which is synthesized via FUT8 expression, were decreased in patients with p53 mutations, compared with those of wild-type p53 [38]. These studies further support the hypothesis that fucosylation is regulated by wild-type p53 function in CRC and other human cancers. Therefore, the possible interaction between aberrant fucosylation and altered p53 function in cancer needs to be examined by future investigation.

Unlike the previous report by Muinelo-Romay et al. [15], we found no association between DFS and the expression of FUT8 in the IHC and microarray cohorts. Nevertheless, high FUT8 protein expression was significantly associated with better DFS when the analysis was restricted to tumors without p53 alteration as a novel finding. Therefore, it is likely that the prognostic effect of FUT8 expression might be selectively confined to the patient subpopulation harboring p53-negative tumors, at least in protein levels. However, this finding was not clearly validated in the microarray cohort, although higher FUT8 mRNA expression showed a nonsignificant trend towards better DFS among patients with p53 wild-type tumors. Those partly inconsistent results between cohorts might be in part due to the technically independent measurement of FUT8 expression based on mRNA or protein levels. In addition, the techniques to detect p53 alterations were different between these cohorts (mutation analysis or IHC). Gene sequencing is the standard for identification of p53 mutations, but still there are methodological issues and the presence or absence of mutation does not directly assess functional activity of p53. The nuclear accumulation of p53 protein by IHC with high-affinity antibodies has been widely used in many clinical studies as surrogate for detecting p53 alterations [33]. Nonetheless, IHC does not recognize null mutations, for instance, inactivated p53 due to nonsense mutations or deletions [32]. Therefore, those methodological disparity for determining the p53 status as well as the lack of standard methods to assess FUT8 expression seem to be the potential limitations of this study. Our cohort also has some limitations since the number of patients in each group were relatively small. Because of the exploratory and retrospective nature of this study, more detailed analysis in a prospective setting is necessary to confirm these results. The present study did not address the biological significance of FUT8, for instance, by using cell or animal models. Although FUT8 gene promotor region is likely to carry the responsive element of wild-type p53 [14], it remains largely unknown if FUT8 expression can functionally affect biological tumor characteristics depending on p53 status. Therefore, mechanisms by which FUT8 levels affect patient outcomes in relation to p53 remain to be elucidated.

In conclusion, the present study utilized two independent cohorts based on microarray or IHC to address the prognostic significance of FUT8 expression in stage II and III CRC, by stratifying patients according to the p53 status. We found that positive staining of FUT8 protein by IHC was significantly associated with better DFS only in tumors with negative p53, despite no association of FUT8 with DFS in positive p53 tumors. Our data partly support the hypothesis that the prognostic value of FUT8 expression is specifically dependent on the p53 status.

## Supporting information

S1 Table Univariate Cox regression analysis for disease-free survival in patients with stage II and III colorectal cancer. (XLSX)

S1 Fig Immunohistochemistry for FUT8 in cultured colorectal cancer cell lines. (PDF)

S2 Fig Immunohistochemistry for FUT8 in colorectal cancer tissues. (PDF)
