# Supplementary figures and images for: Prognostic role of FUT8 expression in relation to p53 status in stage II and III colorectal cancer
Source: PLoS One. 2018 Jul 5;13(7):e0200315. doi: 10.1371/journal.pone.0200315 (PMC6033451; doi:10.1371/journal.pone.0200315)

**S1 Fig. Immunohistochemistry for FUT8 in cultured colorectal cancer cell lines**

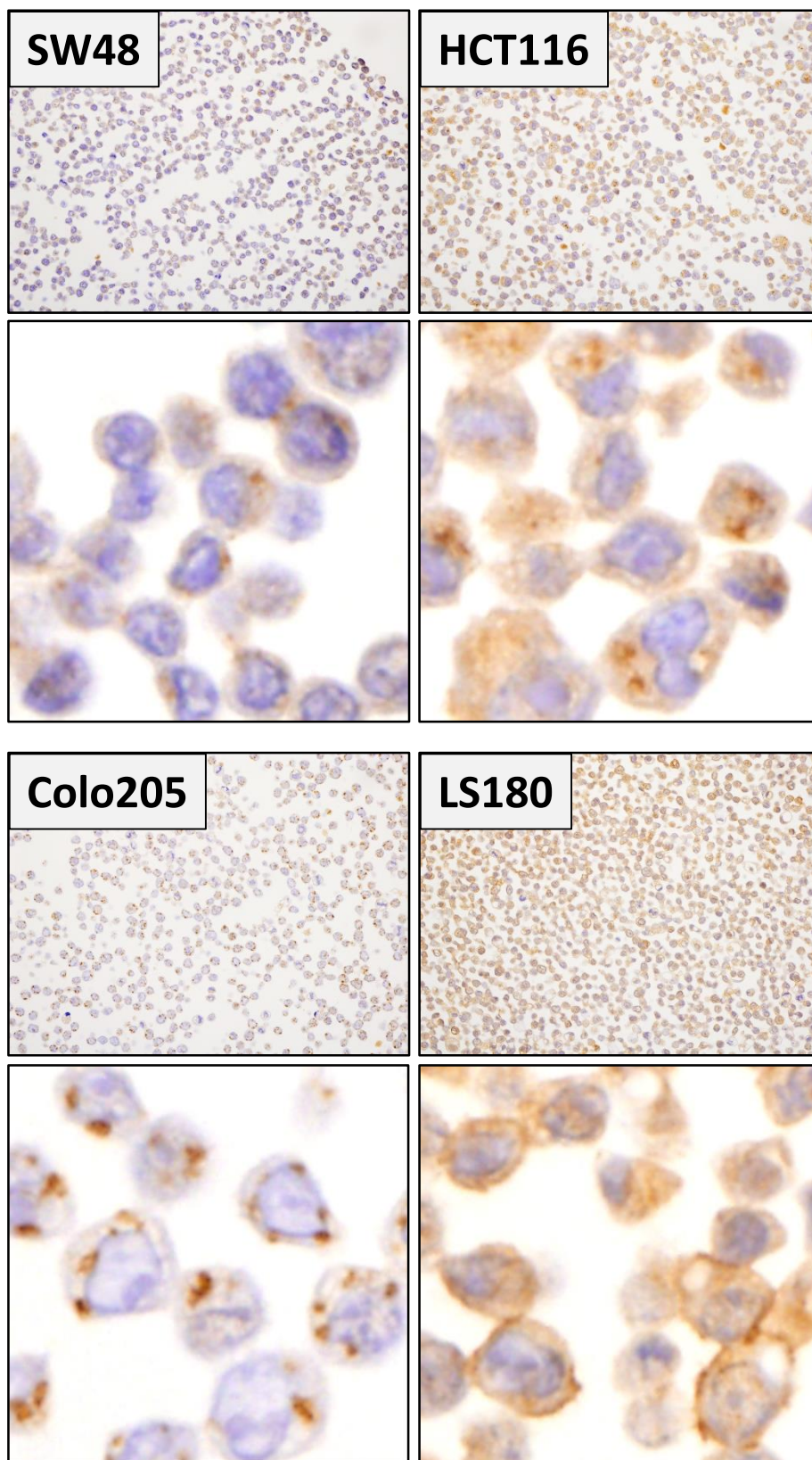

Supplement: S1 Fig — (PDF) [file pone.0200315.s002.pdf]
