# Supplementary material for: Prognostic role of FUT8 expression in relation to p53 status in stage II and III colorectal cancer
Source: PLoS One. 2018 Jul 5;13(7):e0200315. doi: 10.1371/journal.pone.0200315 (PMC6033451; doi:10.1371/journal.pone.0200315)

S2 Fig. Immunohistochemistry for FUT8 in colorectal cancer tissues

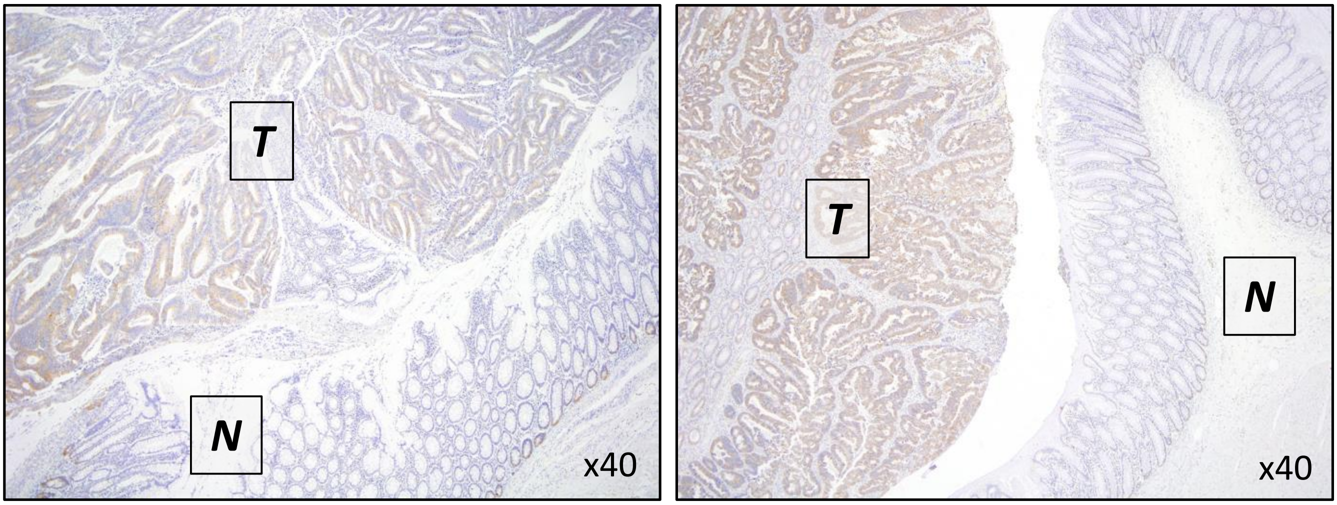

T: colon carcinoma, ***N***: adjacent colon mucosa

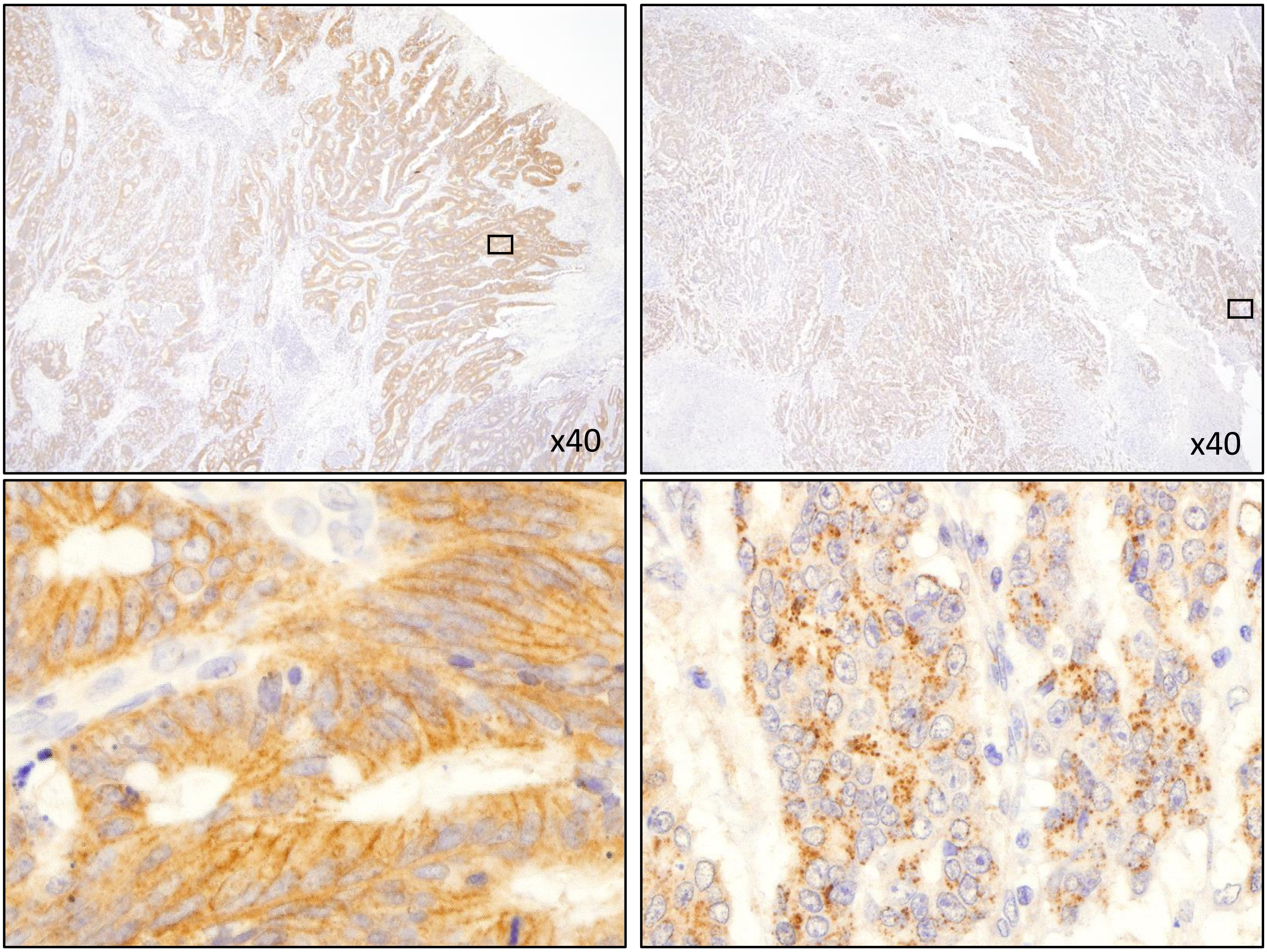

Supplement: S2 Fig — (PDF) [file pone.0200315.s003.pdf]
